# Business Intentions of Australian Veterinary Students—My Business or Yours? A Cluster Analysis

**DOI:** 10.3390/ani13071225

**Published:** 2023-03-31

**Authors:** Adele Feakes, Noel Lindsay, Edward Palmer, Kiro Petrovski

**Affiliations:** 1 School of Animal and Veterinary Science, University of Adelaide, Roseworthy, SA 5371, Australia; 2 Adelaide Business School, University of Adelaide, Adelaide, SA 5000, Australia; 3 School of Education, University of Adelaide, Adelaide, SA 5000, Australia; 4 Davies Livestock Research Centre, School of Animal and Veterinary Science, University of Adelaide, Roseworthy, SA 5371, Australia; 5 Australian Centre for Antimicrobial Resistance Ecology, University of Adelaide, Adelaide, SA 5000, Australia

**Keywords:** entrepreneurial intentions, self-efficacy, entrepreneurship, field of study, identity, nursing, veterinary, students

## Abstract

**Simple Summary:**

Entrepreneurship and intrapreneurship (entrepreneurial behaviour of employees) foster business innovation and growth and support wealth creation and employment in both privately owned and corporate businesses such as those that deliver contemporary veterinary services. Yet we know little about the propensity for entrepreneurship or intrapreneurship of entrants to the veterinary sector. In our study population of veterinary science, entrepreneurship, and human nursing final-year students, veterinary respondents stood out overall for their high entrepreneurial intention and outcome expectations of business ownership but low financial self-efficacy and corporate work intentions. These findings raise questions about (i) the effectiveness of veterinary business curricula which focus on expense management and (ii) the goals and expectations of new entrants to the veterinary sector. However, cluster analysis of responses to these measures revealed that 28.8% of veterinary respondents were entrepreneurial, 17.8% entrepreneurial and intrapreneurial (particularly men), and 13.1% intrapreneurial only (i.e., ~31% intrapreneurial), signalling the opportunities and risks for large veterinary businesses to harness these individuals and their intrapreneurial tendencies. Post hoc analysis revealed cluster differences per veterinary school. To inform curricular change, we recommend further research to evaluate the relative impact of individual factors, admissions factors, and the formal or hidden curricula on entrepreneurial intention in veterinary final-year students.

**Abstract:**

Little is known about veterinary entrepreneurial predisposition. Yet entrepreneurship and intrapreneurship (entrepreneurial behaviour of employees) foster business innovation and growth and support wealth creation and employment in both privately and corporately owned businesses which deliver contemporary veterinary services. We used responses from 515 final-year students in Australian entrepreneurship, nursing, and veterinary programs to capture entrepreneurial intention (EI), outcome expectations (OE-sb), entrepreneurial self-efficacy (ESE), and corporate/large organisation work intentions (CWIs). Veterinary respondents stood out for their high EI and high OE-sb, but low financial ESE and low CWI. Proportions of veterinary, entrepreneurship, and nursing respondents differed markedly across distinct cluster profiles representing entrepreneurial, intrapreneurial, both entrepreneurial and intrapreneurial, indifferent, and corporate employment intentions and attributes. Post hoc analysis revealed proportional cluster membership differences for respondents from different veterinary schools. Our findings raise questions regarding (1) the effectiveness of veterinary business curricula competencies which focus on expense management and (2) the implications of the mismatch of motivations and goals of new veterinary sector entrants whose low intent to work in a corporate environment is at odds with increasing corporate ownership of veterinary practices. To inform curricular change, we recommend further research to evaluate the relative impact of individual factors, admissions factors, and the formal or hidden curricula on entrepreneurial intention in veterinary final-year students.

## 1. Introduction

Entrepreneurship is important for individuals and society, creating jobs and wealth. Entrepreneurship encompasses the development, organisation, and operation of a new business (or business arm) to generate profit, often by solving pressing problems and pain points in a society or customer segment by introducing an innovative product or service or creating new markets. The Vet Futures study, a large international survey of veterinarians, and several other publications highlight the importance of entrepreneurship to veterinarians [1,2,3,4]. Yet we know little about veterinary entrepreneurial or intrapreneurial (entrepreneurial behaviour within a business not owned by them) intentions.

While the process of entrepreneurship has multiple stages, our study was interested in the earliest phase—the pre-inception phase of the entrepreneurial process—the formation of entrepreneurial intention, i.e., “*intent to start a business, to launch a new venture*” [5] (p. 17), still considered an under-researched subject [6]. Upon entry into their chosen study programs, students within their cohorts will be relatively heterogeneous in abilities, experiences, knowledge, motivations, skills, and career aspirations. However, during their studies, learning experiences (vicarious or mastery-based) are likely to meld students’ values and attitudes and their perceptions or real abilities, resource enablers and barriers, further influencing their goals and motivations. Therefore, we expect students from different fields of study to vary in entrepreneurial intention [7].

Gender differences in entrepreneurial intention (EI) levels exist; however, these differences can be accounted for by other antecedents [8,9,10], including a perceived lack of support or the presence of barriers [11]. Descriptive analysis of EI (using a four-item validated scale) [12] of students in five Australian veterinary programs between 2011 and 2014 [13] showed lower EI for women compared to men. However, when the 2014 responses (n = 844) from the same data set were analysed in a hierarchical multiple regression model, accounting for other covariates and attitudinal predictors, gender did not emerge as a strong direct predictor of EI [14]. Thus, our study was more concerned with differences in EI and related constructs between veterinary students and those in other fields-of-study than between genders.

This paper compared the EI of purposefully sampled respondents soon to enter the workforce from three veterinary science programs and two other Australian university-level programs, entrepreneurship and human nursing. We used single items and scale items previously used in student and nascent entrepreneur populations to measure entrepreneurial intent (to start/buy a business, e.g., five years after graduation), entrepreneurial self-efficacy and outcome expectations of entrepreneurship. We present descriptive, bivariate, and cluster analysis, particularly showing the differences in EI of individuals about to enter a veterinary career compared to entrepreneurship or nursing career paths.

Our study contributes to the literature in several ways. First, we compare the level of EI and corporate work intention (CWI) of veterinary final-year students to those in two other fields of study. Second, we provide evidence that entrepreneurial self-efficacy (ESE) and outcome expectations of starting or owning a business (OE-start-business) can be high in individuals who are either high in EI, high in CWI, or high in both. When CWI and ESE are both high, this indicates a propensity for intrapreneurship (entrepreneurial behaviour of employees). Third, as respondents were from three veterinary schools, in a post-hoc analysis, we show ‘school of study’ differences within the ‘field-of-study’, raising further questions about the impact of the veterinary school demographic draw, selection process, or the learning experience.

## 2. Theory, Literature, and Hypotheses

Two theoretical frameworks inform our study. First, the motivation goal sequence (MGS) framework of Locke and Latham (1990) meshed inputs from cognitive and social psychology foundational theories [15]. It proposed that values and motives directly influence goals and intentions, in addition to self-efficacy and outcome expectations. Second, Teixeira and Forte’s (2017) integrated entrepreneurial intentions framework [7] also meshed mechanisms from entrepreneurship theory [16,17], grounded in similar foundational theories to the MGS (i.e., meshed the Theory of Planned Behaviour [18] and the Social Cognitive Theory’s approach to perceived self-efficacy [19]). Teixeira and Forte (2017) acknowledged the contribution of human capital from an individual’s prior exposure to entrepreneurship as separate from that gained during their educational experience (field-of-study). Following the above, a graduate of a tertiary field-of-study would have values, motives, and human capital associated with their prior exposure or family background and then acquire further values, motives, and human capital related to their study experience, vicariously or formally via the curriculum.

### 2.1. Entrepreneurial Intentions and Field-of-Study

Researchers have often regarded educational background as a key demographic and control variable in entrepreneurship studies [20,21]. Exploration of the underpinning mechanisms between disciplinary field-of-study and entrepreneurial intentions is occurring [7,22,23]. For example, Wu and Wu (2008) [23] found that engineering students had higher EI than students of other majors. Teixeira and Forte (2017) [7] contended that such ‘technical’ courses/majors may provide specific know-how that increases the individual’s perceived entrepreneurial self-efficacy, behavioural control, or feasibility, while other courses/majors may affect entrepreneurial intentions via lifting perceived desirability.

Disciplinary field has recently received attention as a community with its own culture or collective identity [24], with values and norms shaping members’ perceptions of the world [25]. For example, in a Netherlands study of a mixed cohort of students (n = 800), differences were found in the perceived status of the entrepreneur among fields of study, with a strong association with individuals’ willingness to become an entrepreneur [26]. In particular, students in economics and business attached a similar status to the entrepreneur as students in the fields of science, technical studies, and humanities. In contrast, students in health and social sciences attached a lower value to the status of the entrepreneur [26]. Furthermore, entrepreneurship study programs are devoted to entrepreneurship topics, in which participation reinforces not only collective identity but also individual and collective (entrepreneurial) self-efficacy (an established driver of entrepreneurial intentions) [27,28]. As such, we proposed the following hypothesis:

**Hypothesis** **1.** *Veterinary science final-year students will be less interested in entrepreneurship than entrepreneurship final-year students.*

The employment environment of the disciplinary field for which the individual trains can broadly dictate attitudes and expectations for self-employment (recognised as a form of entrepreneurship). In well-developed economies, self-employment rates in the service sector tend to be considerably higher than in manufacturing, as relatively modest investments are required to set up most service-providing ventures compared to manufacturing ventures [29]. Most human nursing and veterinary graduates in a developed economy like Australia will work in the service sector, so on this basis one might expect human nursing and veterinary respondents to have similar entrepreneurial intentions.

However, suppose an occupation generates lucrative paid-wage employment under better conditions. In this case, it may decrease the need for self-employment (and, by extension, the formation of EI) of the individuals within it [7]. Within-sector shifts in the availability or not of regular (acceptable) employment options in some fields may make self-employment or new business creation a ‘desirable’ and accepted ‘community’ career path [7,29,30]. In the case of human nursing, the Australian public sector or large corporate companies mostly fund service delivery and nurse employment. While at the time of data collection, privately owned single or multi-site veterinary practices were Australia’s prevalent model of veterinary service delivery [31,32], starting salaries were lower and static for veterinary graduates (AUD 50,000, 2015; AUD 50,000, 2016; AUD 51,600, 2017; AUD 62,600, 2022) compared to nursing graduates (AUD 53,000, 2015; AUD 58,400, 2016; AUD 60,000, 2017; AUD 68,500, 2022), and the total employment outlook was diminishing for veterinary graduates in the pre-COVID-19 pandemic years 2015–2016 (93.0%, 2015; 89.4%, 2016; 87.5%, 2017; 94.7%, 2022) compared to nursing graduates (95.0%, 2015; 93.3%, 2016; 91.7%, 2017; 90.9%, 2022) [33,34,35]. Moreover, given their lower paid-wage, we expect imminent entrants to the veterinary profession to see a greater need for self-employment in order to attain better remuneration. Additionally, because veterinary services are primarily delivered via private enterprise, imminent entrants to the veterinary profession may see business ownership as a route to higher remuneration.

Moreover, discipline-associated training programs can provide an individual with additional human, cognitive, and social capital (social networks and relationships). Various interactions between these capitals can directly affect students’ EI formation while they are undertaking their chosen field-of-study-aligned pathway [7,23]. Veterinary programs are highly technical and intense. They require extensive coursework components and workplace-learning, providing an opportunity to gain human, cognitive, and social capital, and general self-efficacy. Furthermore, Australian veterinary degree study programs are longer than human nursing degree programs (comprising extended bachelor’s or clinical master’s degrees), meaning more time and opportunity for an individual to acquire human, cognitive, and social capital, collective identity norms and attitudes, and self-efficacy. As such, we proposed the following hypothesis:

**Hypothesis** **2.** *Veterinary science final-year students will be more interested in entrepreneurship than nursing final-year students.*

### 2.2. Entrepreneurial, Intrapreneurial, Both or Neither?

While entrepreneurial intention, entrepreneurial self-efficacy, and outcome expectations are likely to vary among respondents, we expect enough similarity such that distinguishable profiles based on similar patterns of responses will emerge. We expect individuals low in entrepreneurial self-efficacy and outcome expectations of business to be low in interest or intention for personally starting or owning a business (non-entrepreneurial). We expect individuals with high entrepreneurial self-efficacy and outcome expectations from entrepreneurship to be high in entrepreneurial intention (potential entrepreneurs). We also expect that some individuals interested in joining corporately owned (large) organisations to use their primary disciplinary skills may also have high entrepreneurial self-efficacy, which could be harnessed by their employers to drive innovation and growth (i.e., entrepreneurial behaviour within a business not owned by them) [33]. Thus, we proposed the following hypothesis:

**Hypothesis** **3.** *Distinct interpretable profiles of differing levels of entrepreneurial intention, entrepreneurial self-efficacy, and corporate work intention will emerge for our study population, such that potential entrepreneurial and intrapreneurial groupings of respondents will be identifiable.*

### 2.3. Prevalence of Profiles per Field-of-Study

To date, diversity in entrepreneurial and intrapreneurial potential across individuals undertaking the veterinary science field-of-study is neither documented nor compared to that in other tertiary study programs. We know that different motivations drive different people towards the same career choices [36,37], but entrepreneurship is often a parallel career choice. When choosing and training for their career paths, veterinarians, like nurses [38,39], are highly motivated to help others. However, veterinarians and veterinary students’ views of professionalism [40], identity role beliefs, and motivations [41] are quite heterogeneous. We expect this to be reflected proportionately across distinct profiles representing different combinations of entrepreneurial intentions, entrepreneurial self-efficacy, and corporate work intentions. Thus, we proposed the following hypothesis:

**Hypothesis** **4.** *The proportional representation of identified cluster-based profiles of entrepreneurial intention and self-efficacies will differ for veterinary respondents to entrepreneurship or nursing respondents in our study population.*

## 3. Methods

### 3.1. Study Population and Data Capture

This research utilised data collected as part of a comprehensive study conducted with the collaboration of staff at three Australian universities. Data were collected voluntarily from respondents as close as possible to the end of their university program. For some students, this was in the last weeks, while for others, it was in the first weeks of the final year, the only time the whole cohort was together. The study was approved by each participating university’s Human Ethics Research Committees.

Responses analysed and reported in this paper were collated from questionnaire items regarding entrepreneurial and work intention. We piloted the survey with volunteer academics, veterinarians, and students not involved in the study. Data capture involved paper-based surveys during on-campus contact time.

Veterinary students invited to participate in the survey were all final-year enrolled veterinary students from three Australian veterinary schools (University 1—2015, 2016; University 2—2015, 2016; University 3—2016), totalling 315 enrolled students, of which 274 responded, for a response rate of 87%. The 274 veterinary student respondents represented 23% of the 1213 Australian final-year veterinary students for 2015 and 2016 (593 and 620, respectively). Non-veterinary students surveyed were in their last semester in entrepreneurship and nursing programs at University 1 in 2015 and 2016. Entrepreneurship respondents numbered 98 (60%) of 162 enrolled; nursing respondents numbered 201 (75%) of 266 enrolled.

We undertook statistical analysis using IBM SPSS Statistics 28^®^ software for Windows^®^ (SPSS, Inc., Chicago, IL, USA) and MPlus v8.7 [42]. We analysed 515 of the 573 responses (89.9%), excluding responses deemed spurious or with data missingness >5% for all key variables. The final data set (n = 515) had missingness deemed as MCAR (missing completely at random) with <5% variables with missing data (Little’s test *p* = 0.008). Table 1 provides a demographic summary of respondents whose data we analysed for this study.

### 3.2. Measures

#### 3.2.1. Entrepreneurial and Corporate Work Intention

We used single items to measure entrepreneurial intention (EI) and intention to work in the corporate field (CWI). Our measure of entrepreneurial intention was operationalised from measures used in prior seminal entrepreneurship studies incorporating conceptions of starting or buying into a business as pathways into venture ownership and development, and degree of conviction (time frame) [16,43,44]. Conversely, we operationalised our measure of corporate work intention to capture respondents’ propensity to see themselves working (i.e., in a paid role) with a corporate organisation (an established large company). We asked respondents to rate their level of intent for each of the following statements:

*“Do you intend to start or buy into a business in the foreseeable future, e.g., within five years of graduation?”*

*“Do you intend to work in the corporate field (i.e., a large company)?”*

We captured responses to these two stand-alone measures on unipolar 10-point scales where 1 was ‘no intention’ and 10 was ‘complete intention’. We rescaled answers for these two items to a range of one to seven (to align with the Likert-like response range of the Entrepreneurial Self-Efficacy items) [45], after which we calculated mean EI and mean CWI.

#### 3.2.2. Outcome Expectations

The MGS and the integrated entrepreneurial intentions framework each include a construct related to outcome expectations as antecedents to intentions. Value expectation constructs in Teixeira and Forte’s (2017) integrated entrepreneurial intentions framework originate from the attitude toward the behaviour [18] or perceived desirability (a person’s value expectations of business outcomes) [16,46]. The expectancy construct in the MGS model originates from Vroom’s Expectancy Theory [47]. We used the three-indicator perceived desirability measure “*In general, starting a business would be for me…worthless-worthwhile; disappointing-rewarding; negative-positive*” to represent outcome expectations. This measure required a response on a unipolar rating scale from one to ten [45]. The data reliability of the three items was highly satisfactory (Cronbach’s alpha 0.96), as were the AVE and composite reliability (0.9 and 1.0, respectively), showing the three indicators related well to our respondents. Responses for these three items were rescaled to a range of a maximum of seven [48], after which we computed factor scores as composites (summed and averaged) for outcome expectations-start-business (OE-start-business).

#### 3.2.3. Entrepreneurial Self-Efficacy

We used the 19 indicators of McGee et al.’s (2009) [45] multidimensional entrepreneurial self-efficacy scale (m19ESE) to measure self-efficacy in venture creation process activities (see Table 2): (a) searching, planning, and marshalling activities necessary in the pre-venture phase (ESE-pre-venture); (b) people implementation activities (ESE-people), and (c) financial implementation activities (ESE-Financial). We asked respondents to answer, *“How much confidence do you have in your ability to: Design a product or service that will satisfy customer needs/wants”* and so on (see Table 2) using a unipolar seven-point Likert-like response format where 1 = none at all and 7 = very much. For our data, the scales’ reliability was highly satisfactory (Cronbach’s alpha = 0.95; McDonald’s omega = 0.95) as a full scale, and for each of the three subdimensions, Cronbach’s alpha and McDonald’s omega were all >0.90. The average variance extracted and composite reliabilities for the three subdimensions were all >0.5 and >0.9, respectively, using standardised factor loadings from a satisfactorily fitting CFA measurement model also incorporating the outcome expectations latent variable. We computed factor scores as composites (summed and averaged) for ESE-pre-venture, ESE-people, and ESE-Financial.

#### 3.2.4. Field-of-Study

We obtained multi-categorical data on participants’ ‘field-of-study’ (discipline-based study programs) and institutions (universities), which we transformed into three separate dichotomised variables representing fields-of-study and five different dichotomised variables to separate the three veterinary cohorts.

### 3.3. Data Preparation and Statistical Analysis

We replaced missing data by applying the E.M. procedure in SPSS before analysis. We checked that skewness and kurtosis were acceptable. We compared means using the ANOVA procedure, using the Dunnett t (2-sided) post hoc test when variances were homogenous and the Games–Howell post hoc test when variances were non-homogenous. The significance of differences between groups is reported at *p* < 0.05 unless otherwise indicated. To establish the face validity and discriminatory validity of the latent variables (ESE and OE-start-business), we used confirmatory factor analysis with robust maximum likelihood (MLR). We used composite factor scores for all measures using latent variables.

We made no assumptions of causal relationships between variables for our study population.

## 4. Results

### 4.1. Correlations

Correlations between the main variables of interest appeared logical (Table 3). EI and CWI were weakly correlated. The ESE and OE-start-business variables positively correlated with EI, while the ESE variables only weakly correlated with CWI, and OE-start-business did not at all.

Being a woman respondent negatively correlated with age and all variables related to entrepreneurship but strongly correlated with being a nursing respondent. Being older was negatively correlated with being a nursing respondent but positively correlated with being a veterinary respondent (*r* = 0.31), OE-start-business (*r* = 0.28), ESE-pre-venture (*r* = 0.22), and EI (*r* = 0.32).

Being an entrepreneurship respondent positively correlated with international enrolment status (*r* = 0.69), all three entrepreneurial self-efficacies (*r* = 0.35, *r =* 0.15, and *r* = 0.30, respectively), OE-start-business (*r* = 0.24), EI (*r* = 0.31), and CWI (*r* = 0.34).

Being a nursing respondent negatively correlated with all variables related to entrepreneurship and age.

Being a veterinary respondent negatively correlated with CWI (*r* = −0.30) and ESE in implementing finances (*r* = −0.09) but positively correlated with OE-start-business (*r* = 0.19) and EI (*r* = 0.22).

### 4.2. Mean Comparisons

**Figure 1:**
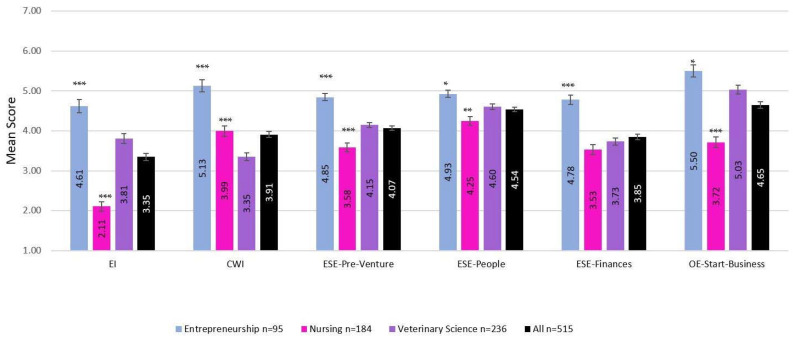
Mean scores (with standard error bars) of entrepreneurial intention (EI), intention to work in large (corporate) organizations (CWI), entrepreneurial self-efficacies (ESE) (pre-venture, implementing people and implementing finances dimensions), and outcome expectations (OE) of starting/owning a business mean scores for respondents in each of the three fields-of-study and for the whole study population (n = 515). Significant difference of veterinary respondents to other disciplines * *p* < 0.05, ** *p* < 0.01, *** *p* < 0.001.

Table 4 and Figure 1 provide mean comparisons for respondents per field-of-study and gender for entrepreneurial and corporate work intentions.

Overall, respondents indicated higher mean levels of CWI than EI.

Differences between groups were confirmed using ANOVA. Respondents in the three different fields-of-study differed in mean EI (*F*_(2,514)_ = 77.914, *p* < 0.001) and CWI (*F*_(2,514)_ = 41.941, *p* < 0.001). Veterinary respondents had the lowest CWI (*p* < 0.001) compared to all other respondents. Veterinary respondents were lower in EI than entrepreneurship respondents; therefore, Hypothesis 1 was supported. Veterinary respondents were higher in EI than nursing respondents; therefore, Hypothesis 2 was supported.

Overall, women respondents were lower than men in EI (*F*_(1,514)_ = 54.462, *p* < 0.001) and CWI. (*F*_(1,514)_ = 6.051, *p =* 0.014) (Table 5). In contrast, within each of the three fields of study, there was no difference between women and men respondents for CWI, and within entrepreneurship and nursing respondents, there was no difference between women and men for EI However, among veterinary respondents, women were of lower EI than men (*F*_(1,234)_ = 18.724, *p* < 0.001).

Figure 1 and Table 5 summarise the mean factor scores of the established antecedents of entrepreneurial intention: entrepreneurial self-efficacy in pre-venture activities such as ‘searching’, ‘planning’, and ‘marshalling’ (ESE-pre-venture), in ‘implementing people’ (ESE-people), in ‘implementing finances’ (ESE-finances) start-up operational activities, and outcome expectations of starting or owning a business outcome expectations (OE-start-business). Overall, respondents rated themselves around the neutral position for ESE-pre-venture (*u* = 4.07) and ESE-finances (*u* = 3.85) and higher for ESE in implementing people (*u* = 4.54) and for OE-start-business (*u* = 4.65) (n = 515).

Differences between field-of-study groups and genders were confirmed using ANOVA. Between group differences identified for respondents in different fields-of-study differed in mean ESE-pre-venture (*F*_(4,610)_ = 25.473, *p* < 0.001), ESE-people (*F*_(4,610)_ = 7.231, *p* < 0.001), ESE-finances (*F*_(4,610)_ = 15.933, *p* < 0.001), and OE-start-business (*F*_(4,610)_ = 26.199, *p* < 0.001). Respondents of different genders differed in mean ESE-pre-venture (*F*_(1,613)_ = 46.830, *p* < 0.001), ESE-people (*F*_(1,613)_ = 10.223, *p* < 0.01), ESE-finances (*F*_(1,613)_ = 46.860, *p* < 0.001), and OE-start-business (*F*_(1,613)_ = 37.644, *p* < 0.001).

Nursing respondents rated themselves lowest across all three ESE measures and for OE-start-business. Veterinary respondents rated themselves similarly to nursing respondents for ESE-finances but rated themselves higher for ESE-pre-venture, ESE-people, and OE-start-business. Veterinary respondents rated themselves lower than entrepreneurship respondents for ESE-pre-venture and ESE-finances but were not significantly different from entrepreneurship respondents for ESE-people and OE-start-business. Veterinary respondents’ self-reported ESE-pre-venture, ESE-people, and their perceived value (outcome expectations) of starting a business were at or above the study population mean and for OE-start-business, second only to entrepreneurship respondents.

Overall, women respondents rated themselves lowest for all four measures—the three ESE variables and OE-start-business. However, respondents within their fields-of-study cohorts did not rate themselves differently for ESE-people (see Table 5). Within the entrepreneurship field-of-study cohort, gender differences were only in OE-start-business; within the nursing cohort, gender differences were only in ESE-pre-venture. Within the veterinary cohort, women rated themselves lower than men on all three other measures—ESE-pre-venture, ESE-finances, and OE-start-business.

### 4.3. Post Hoc Mean Comparisons per Veterinary School

**Figure 2:**
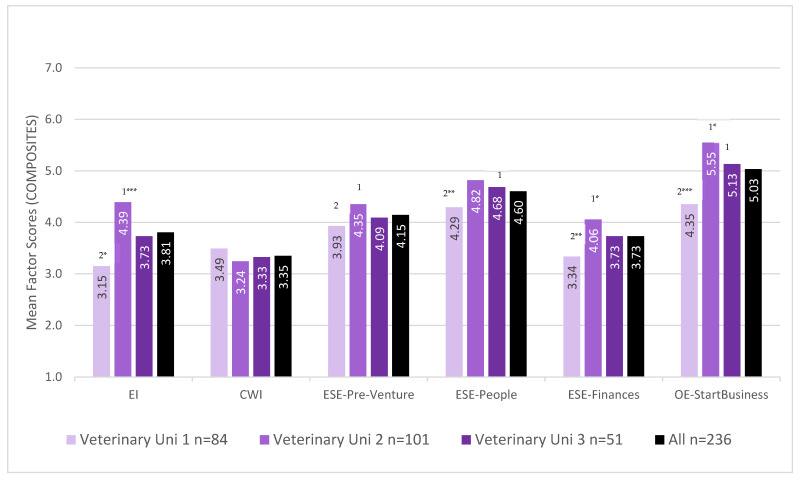
Mean scores (with standard errors) of entrepreneurial intention (EI), corporate work intention (CWI), entrepreneurial self-efficacy (ESE) (Pre-Venture, People and Finances dimensions), and outcome expectations of starting a business (OE-start-business), of respondents in the three participating veterinary schools (n = 236). Significant difference of respondents of University 1 to the other two veterinary schools (* *p* < 0.05, ** *p* < 0.01, *** *p* < 0.001) ^1^ University 1, ^2^ University 2, ^3^ University 3.

We compared all our variables of interest for veterinary respondents at the three veterinary schools represented in our study population (Figure 2).

Mean score comparison using the ANOVA procedure showed no between-group main effect for CWI (*F*_(2,233)_ = 0.669, *p* = 0.513). Between-group effects were found for EI (*F*_(2,233)_ = 10.429, *p* < 0.001), ESE-pre-venture (*F*_(2,233)_ = 6.576, *p* = 0.002), ESE-people (*F*_(2,233)_ = 7.280, *p* < 0.001), ESE-finances *F*_(2,233)_ = 6.950, *p =* 0.001), and OE-start-business (*F*_(2,233)_ = 14.671, *p* < 0.001).

Overall, veterinary respondents from the three veterinary programs did not differ for the CWI measure. Veterinary respondents of University 1 self-rated themselves lower for EI, all three ESE and OE-start-business measures than respondents of University 2. Veterinary respondents of University 1 and 3 were similar in EI and all three ESE measures, but veterinary respondents of University 1 self-rated themselves lower for OE-start-business.

Table 6 illustrates the diversity in the timing and content of learning activities relevant to the ‘in professional life ‘attribute group ‘An Understanding of the Business of Veterinary Practice’ [49] (p. 41) across the three veterinary programs of the veterinary respondents in our study population at the time of data collection (2015–2016) [50].

University 3 respondents had formal curricular content about veterinary business early in their veterinary studies; University 1 veterinary respondents had formal curricular content about veterinary business early-midway in their veterinary studies; while University 2 respondents had formal curricular content learning and teaching at the latest point in their veterinary study programs.

### 4.4. Cluster Analysis

To test Hypothesis 3, we undertook K-means clustering of the intention measures (EI and CWI) and the ESE measures (ESE-pre-venture, ESE-people, ESE-finances). The K-means clustering procedure revealed five interpretable groups, with the smallest cluster at 10.1% (i.e., acceptably greater than 5% of the study population) and the largest cluster at 26.5%. We did not include outcome expectations in the K-means clustering procedure as we were most interested in how entrepreneurial self-efficacy related to intentions for entrepreneurship compared to working with a corporate (large) organisation. However, we report the mean score of outcome expectations of starting a business (OE-start-business) for each cluster profile. ANOVA analysis confirmed significant differences between clusters for EI (*F*_(4,510)_ = 330.893, *p* < 0.001), for OE-start-business (*F*_(4,510)_ = 69.805, *p* < 0.001), for ESE-pre-venture (*F*_(4,510)_ = 131.997, *p* < 0.001), for ESE-people (*F*_(4,510)_ = 93.141, *p* < 0.001), for ESE-finances (*F*_(4,510)_ = 117.715, *p* < 0.001), and for CWI (*F*_(4,510)_ = 244.647, *p* < 0.001).

Cluster Profile 1 (n = 92, 17.9%) identified respondents with scores above the population mean of the total sample for EI, ESE-pre-venture, ESE-people, and ESE-finances. Still, scores were below the population mean for CWI. This group also had OE-start-business scores well above the mean of the total sample. We named this group ‘entrepreneurial’.

Cluster Profile 2 (n = 123, 23.8%) identified respondents with scores above the mean of the total sample for EI, ESE-pre-venture, ESE-people, ESE-finances, and CWI. This group had OE-start-business scores above the population mean. This group was ‘both’ entrepreneurial and intrapreneurial.

Cluster Profile 3 (n = 106, 20.6%) identified respondents with scores below the mean of the total sample for EI, around the mean for ESE-pre-venture, and above the mean ESE-people, ESE-finances, and CWI. This group had OE-start-business scores below the mean of the full sample. We named this group ‘intrapreneurial’.

Cluster Profile 4 (n = 54, 10.5%) identified respondents with scores well below the mean of the total sample for EI, ESE-pre-venture, ESE-people, and ESE-finances but above the mean for CWI, with very low OE-start-business scores. This group was neither entrepreneurial nor intrapreneurial but had a positive intention to work in a large organisation. We named this group ‘corporate/large organisation employment’.

Cluster Profile 5 (n = 140, 27.2%) identified respondents with scores below the mean of the total sample for each of the measures: EI, the three ESEs, CWI, and OE-start-business. Respondents of this cluster were neither entrepreneurial nor intrapreneurial and had no distinct intention to work in a large organisation. We named this group ‘indifferent’.

**Figure 3:**
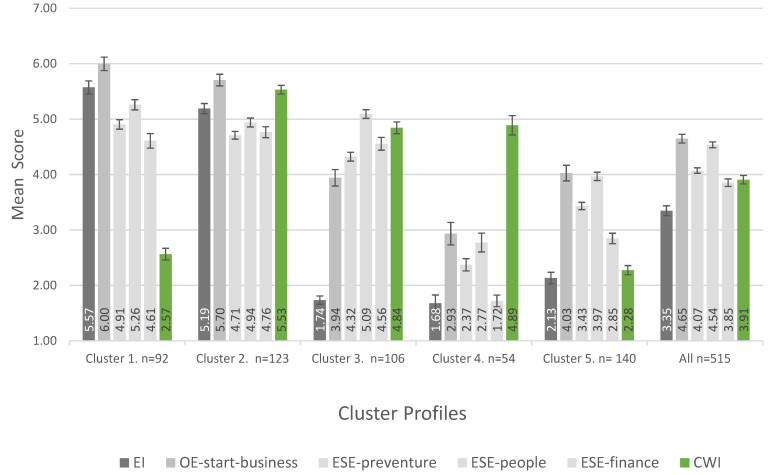
Attributes of cluster profiles—Mean scores (with standard errors) of entrepreneurial intention (EI), outcome expectations of starting a business (OE-start-business), entrepreneurial self-efficacy (ESE) (Pre-Venture, People and Finances dimensions), and corporate work intention (CWI) of respondents in different cluster profiles and for the whole study population (n = 515).

Figure 3 illustrates the mean scores of EI, entrepreneurial self-efficacies (ESEs), outcome expectations in starting a business (OE-start-business), and corporate work intention (CWI) for respondents of each cluster profile. The distinctness of these interpretable cluster profiles provides support for Hypothesis 3.

### 4.5. Cluster Profile Representation per Field-of-Study

**Figure 4:**
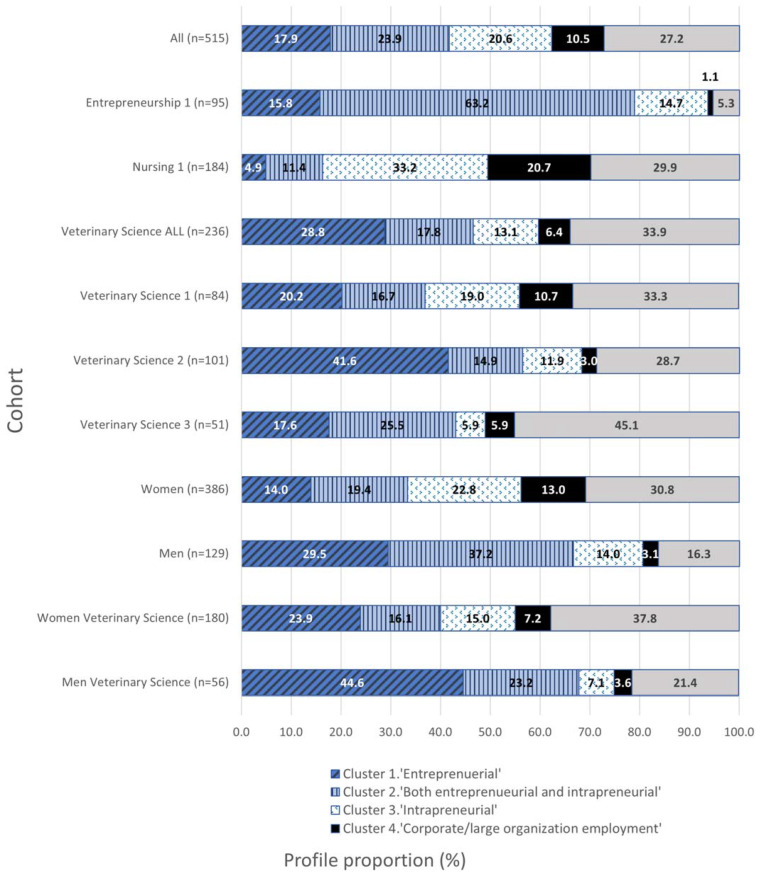
Profile proportions per field of study, veterinary school, and gender.

To test Hypothesis 4, we undertook frequency analysis of the five cluster profiles that emerged in the K-means cluster procedure (Figure 4).

Cluster Profile 5 (‘indifferent’) was much less common in entrepreneurship respondents (5.3%) but more common in veterinary respondents (27.2%) and nursing respondents (29.9%).

Cluster Profile 4 (‘corporate/large organisation employment’) was less common in entrepreneurship respondents (1.1%) than veterinary respondents (10.5%) and more common in nursing respondents (20.7%).

Respondents in Cluster Profiles 1 (‘entrepreneurial’), 2 (both ‘entrepreneurial + intrapreneurial’) and 3 (‘intrapreneurial’) all had higher than mean ESEs. Cluster profiles 1 (‘entrepreneurial’) and 2 (‘both’) were the profiles identified as having higher than mean ESEs and EI Nursing respondents were the least represented, while entrepreneurship respondents were the most represented across Cluster Profiles 1, 2, and 3 combined. Similarly, nursing respondents were less common, while entrepreneurship respondents were the most common across Cluster Profiles 1 and 2 combined.

Cluster Profile 3 (‘intrapreneurial’) was less common in veterinary (20.6%) and entrepreneurship (14.7%) respondents and more common in nursing respondents (33.2%).

Cluster Profile 2 (both ‘entrepreneurial + intrapreneurial’) was less common in nursing respondents (11.4%) and most common in entrepreneurship respondents (63.2%).

Cluster Profile 1 (‘entrepreneurial’) was less common in nursing (4.9%) cohorts but most common in veterinary respondents (28.8%), particularly those in University 2 (41.6%) or men veterinary respondents (44.6%).

Five interpretable profiles emerged for the sample population based on entrepreneurial intentions, entrepreneurial self-efficacy, and intentions to work in large organisations. These cluster profiles differed in the proportional representation of final year students of entrepreneurship, nursing, and veterinary fields of study, and for veterinary respondents from different veterinary schools and women and men veterinary respondents. Overall, the diversity in the proportional representation of the cluster profiles of veterinary and other occupational cohorts supports Hypothesis 4.

Table 7 summarises the hypotheses underpinning this research and the level of support for each.

## 5. Discussion

In this study, we focused on the effect of being a respondent in the veterinary field. Our primary objective was to gain insights into the mindsets affecting business intentions and motivations of prospective (veterinary) entrepreneurs. In doing so, we highlighted that field-of-study appeared to be associated with the level of entrepreneurial intention (EI) of future (veterinary) entrepreneurs alongside the theoretical and long-established antecedents—outcome expectations and entrepreneurial self-efficacy. This was in line with the MGS axis of goal-setting theory [15] and the integrated entrepreneurial intentions framework of Teixeira and Forte (2017) [7]. That level of EI can vary per field-of-study is relevant to educators and policy makers who may need to consider such nuances when promoting entrepreneurship or intrapreneurship inclusion in curricula.

We compared veterinary, nursing, and entrepreneurship students, expecting the latter to be highest in entrepreneurial attitudes, cognitions, and intentions because of self-selection and educational inputs [51,52]. As expected, entrepreneurship respondents were the highest in all EI, ESE, and OE-start-business measures. Veterinary respondents were lowest for corporate work intentions but second highest after entrepreneurship respondents for EI Besides veterinary respondents, the EI of the entrepreneurship and nursing respondents in our study population was lower than their corporate work intention (CWI). The time frame included in the EI single item measure “Do you intend to start or buy into a business in the foreseeable future, e.g., within five years of graduation?” may have influenced the lower EI more than CWI.

Overall, associated with their field-of-study, students did vary in their level of the known antecedents to EI. Veterinary respondents self-rated themselves between entrepreneurship and nursing respondents for ESE in pre-venture activities and ESE-people, but similar to nursing respondents in low ESE-finances. That veterinary respondents of University 2 self-rated themselves much higher in EI than veterinary respondents of University 1 or University 3 was consistent with the findings reported for early, mid-, and late-program veterinary respondents from five universities in a prior study (n = 844) [14] using a four-item entrepreneurial intention scale [12]. Similar to field-of-study, the (veterinary) school of study variable may capture acquired collective identity norms about business and entrepreneurship and differences in the beliefs and attitudes of respondents towards entrepreneurship, other than OE-start-business and ESE. The higher ESE and OE-start-business of veterinary respondents from University 2 may also be related to greater levels of experience in business or farming of these respondents, human capital, family background social norm effects, or higher resilience or risk-taking in rural background respondents.

### 5.1. Implications for the Veterinary Profession and Animal Healthcare Sector

A concerning implication for the veterinary sector is that veterinary respondents appeared more strongly motivated by OE-start-business rather than by feelings of capability in entrepreneurial tasks, particularly financial. In that case, the ESE they bring with them may be insufficient, as self-efficacy (confidence in one’s capability) informs competency [19,53,54] as well as intentions [18]. Thus, they may not bring a ‘balanced’ skillset or sufficient self-confidence to their business [54]. Furthermore, their lack of confidence risks avoidance behaviours in critical entrepreneurial activities [54]. That veterinary respondents’ ESE-finances are comparatively low while their EI levels were comparatively high signalled a mismatch in goals and values. The private veterinary practice sector appears to make an insufficient profit, reflected in the lower remuneration of its professionals compared to other disciplines [35,55].

A positive implication arose from our results for veterinary business or practice owners undertaking succession planning, where veterinary respondents indicated a high intent to start or buy into a business. However, this may backfire on the profession. The current era of corporate purchase of ‘retiring’ baby boomer-owned practices at high multiples of ‘EBITDA’, has translated into a lack of availability of established practices for purchase or part-purchase. As such, the propensity of the next generation of veterinarians to start a practice may then be expressed in ‘start-ups’ rather than buying into existing practices. To gain clientele, start-ups typically run on extended consulting hours, low staff:workload ratios, and provision of after-hours services (if rural or regional). These are known factors in decreased job satisfaction, burnout, and eventual attrition in the veterinary sector [56,57].

The lower mean EI for women veterinary respondents, in particular, is of concern. Gender differences in entrepreneurial intent are highly relevant considering the major gender shift of the veterinary profession towards the majority being women [55,58,59,60,61]. Based on Australian data, most veterinarians are women (approximately 66%), and most Australian graduates are women (about 75%); thus, it is likely that a high proportion of future veterinary entrepreneurs should be women. However, low EI and low ESE-finances in women veterinarians are likely to impact private practice ownership levels and profitability (the latter is important for sustainability and growth). This signals an imperative for further research to establish the source of the gender differences in EI for veterinary respondents. In particular, further research is warranted to evaluate if the hidden (e.g., role modelling) and taught curriculum of veterinary programs could be harnessed to redress this issue.

Lastly, the results of our study have implications for corporately owned or very large privately owned, multi-site veterinary practices. On the one hand, we found a starkly lower interest of veterinary respondents compared with the entrepreneurship and nursing respondents to work in the corporate field. On the other hand, we note that Clusters 2 (both entrepreneurial and intrapreneurial) and 3 (intrapreneurial) were well represented by veterinary respondents (17.8% and 13.1%, respectively, around 31% overall). Younger employees are more likely to engage in nascent intrapreneurship (entrepreneurial behaviour within a business not owned by them) than nascent entrepreneurship [62]. Thus, for large or corporately owned veterinary enterprises, this presents an opportunity to harness these individuals and their entrepreneurial tendencies [62], but with the warning that veterinary respondents, particularly men, of Cluster 2 (high EI, high ESE, high CWI), may also be inclined to leave to pursue their own EI.

### 5.2. Implications for Educational Practice

We found that entrepreneurship, nursing, and veterinary respondents, i.e., quite different fields of study, differed in EI. We also confirmed that veterinary respondents from different veterinary programs differed in EI, similar to those previously reported for the same universities [14]. The difference has implications for educators, as it may be rooted in the differing attitudes of students regarding business and entrepreneurship brought with them upon entry into the veterinary program because of their background and human capital. Alternatively, the difference may be due in part to exposure to an explicit or hidden curriculum (e.g., how and when students are exposed to business teaching/learning) as a student’s beliefs or attitudes can change because of exposure to formal or informal entrepreneurship education or experience.

While current veterinary curricula accreditation bodies mandate the teaching of business and financial competencies, in our study, veterinary respondents self-rated themselves as low in financial self-efficacy—suggesting that teaching does not necessarily equal learning. When considering entrepreneurship content (also known as business content) for veterinary or other accredited professional program curricula, educators will be guided first by their accrediting body’s requirements. The current U.S., U.K., and Australian accrediting bodies’ requirements for veterinary curricula are limited in their focus on ‘economic’, financial, and performance indicators, management, fee calculation, income, overheads, and expenditure in running veterinary businesses. There is no mention of how to grow a (veterinary) business’s top line via entrepreneurship to sustain and grow the business and its people, despite scholars and national professional bodies calling for entrepreneurship in the veterinary sector [1,2,63,64].

Our study is important to educators and policy makers when deciding on resource allocation and pedagogical approaches to entrepreneurship content within or alongside discipline-specific programs such as veterinary science. When entrepreneurship content must sit alongside the content of a primary discipline, initiatives to build entrepreneurial mindsets and capability in tertiary students should be carefully considered, so they are ‘not impaled’ [22] by pedagogy malaligned with student cognitions and motivations.

### 5.3. Limitations and Future Research

This study has some limitations. These include the disproportion in the gender representativeness in the study population, the convenience sample, and covering only three of the seven Australian universities with veterinary schools. The non-veterinary respondents were from only one of these three universities. Also of note is that the domestic-to-international student profile of entrepreneurship students differed from those of the other disciplines, which may have introduced a response bias.

Other limitations include data collection being at a single point and respondents being close to graduating rather than graduated. However, it is well established that intentions are strong predictors of eventual behaviour [18]. We purposefully sought participants with imminent employment decision points regarding (re)entering further study, employment, self-employment (sole trader), or self-employment (entrepreneurship) [16]. We purposefully sought participants in helping and non-helping vocations [65,66] for expected variance in our study constructs and those from specific disciplinary cohorts for precision via within-group response homogeneity. Our respondents were 23% of the Australian veterinary final year cohort at the sampling time, so they can be considered representative. To investigate the field-of-study effect on entrepreneurial intentions, future research is needed with more participants from a single institution across a greater number of study programs (e.g., 20 or more) to enable multi-level modelling to separate field-of-study effects. A longitudinal study within a field-of-study, such as veterinary science, may be appropriate to differentiate the impact of field-of-study and personal attributes or perhaps a mixed-methods study incorporating detailed qualitative data on the curriculum and students’ perceptions of the real and hidden curriculum.

All three participating veterinary schools had different admissions procedures and different veterinary business curricula timing for the cohorts in the study population (and still do). While our results indicated an effect of (veterinary) school-of-study, our study is limited in that we could not ascertain or pinpoint how the impact was associated with the veterinary schools’ admissions procedures, demographic draw, or curricula, taught, or hidden. We suggest further research and more sophisticated analyses of the effect of the interrelations and origins of the difference between veterinary schools on veterinary students’ EI.

## 6. Conclusions

Our key message for veterinary educators and research scholars is the need for further research to evaluate the relative impact of individual and group level factors (e.g., gender role beliefs, values and beliefs associated with field-of-study collective identity formation, hidden or formal curricula) on entrepreneurial intention in veterinary final-year students. Such research would aim to mitigate the negative and capitalise on the positive mechanisms to inform curricula change.

We have two key messages for the veterinary sector. First, Australian veterinary final-year students in our sample population were highly entrepreneurial. However, they had relatively higher outcome expectations of business ownership than confidence in their capability to undertake entrepreneurial tasks, particularly financial tasks, despite the focus of veterinary curricula standards on financial acumen. As confidence is positively associated with competence, such entrants to the veterinary profession may bring an ‘unbalanced’ skillset or ‘avoidance tendencies’ regarding financial acumen to their business ‘entre’.

Our second key message for the veterinary sector is that 17.8% of the Australian veterinary final-year students in our sample population were both ‘entrepreneurial and intrapreneurial’ (particularly men) and 13.1% intrapreneurial only (i.e., ~31% intrapreneurial). This signals the opportunity (and risk) for large veterinary businesses to harness (or fail to engage) this intrapreneurial propensity.

## Figures and Tables

**Table 1:** Demographic profile of respondents after data hygiene (n = 515).

	Total	Median Age Years	Male % (n)	Female % (n)	International * Enrolment %
Entrepreneurship	95	24.0	57.9% (n = 55)	42.1% (n = 40)	78.9%
Nursing	184	21.0	9.8% (n = 18)	90.2% (n = 166)	6.5%
Veterinary Science (all)	236	24.0	23.7% (n = 56)	76.3% (n = 180)	8.1%
Veterinary Sc Uni1	84	24.0	22.6% (n = 19)	77.4% (n = 65)	7.1%
Veterinary Sc Uni2	101	23.0	25.7% (n = 26)	74.3% (n = 75)	0.0%
Veterinary Sc Uni3	51	24.0	21.6% (n = 11)	78.4% (n = 40)	25.5%
Total	515	24.0	25.0% (n = 129)	75.0% (n = 386)	20.6%

* 21% of 2015–2016 Australian veterinary program final-year students were international students (A.V.A., 2017).

**Table 2:** Indicators used to form factor scores for ESE-pre-venture, ESE-people, and ESE-financial subscales and the unidimensional scale for business outcome expectations [45].

Factor	Item	Factor Indicators
Outcome-Expectations -Starting/owning a business	OE1.	Worthless (1)… (10) worthwhile
	OE2.	Disappointing (1)… (10) rewarding
	OE3.	Negative (1)… (10) positive
ESE-Preventure	E1.	Brainstorm (come up with) a new idea product/service
	E2.	Identify the need for a new product or service
	E3.	Design a product or service that will satisfy customer needs/wants
	E4.	Estimate customer demand for a new product or service
	E5.	Determine a competitive price for a new product or service
	E6.	Estimate the amount of start-up funds and working capital
	E7.	Design effective marketing/advertising-new product or service
	E8.	Get others to identify with/believe in my vision/plan-new business
	E9.	Network, i.e., make contact with/exchange information with others
	E10.	Clearly/concisely explain verbally/in-writing my business idea
ESE-People	E11.	Supervise employees
	E12.	Recruit and hire employees
	E13.	Delegate tasks/responsibilities to employees in my business
	E14.	Deal effectively with day-to-day problems and crises
	E15.	Inspire, encourage, and motivate my employees
	E16.	Train employees
ESE-Finances	E17.	Organise and maintain the financial records of my business
	E18.	Manage the financial assets of my business
	E19.	Read and interpret financial statements

**Table 3:** Correlations (Spearman’s Rho) of demographic, self-report single items and composite factor scores (n = 515). (Significant correlations ≥0.3 in bold).

		Mean	SD	1	2	3	4	5	6	7	8	9	10	11	12	13
1	Gender (1 = women)	0.75	--	--												
2	Age (Winsorized to 32 years)	23.97	3.22	−0.23**	--											
3	Parents Business/farmed (1 = yes)	0.62	--	0.01	0.00	--										
4	Self-Business/farmed (1 = yes)	0.14	--	−0.02	0.13**	0.10*	--									
5	International (1 = yes)	0.21	--	−0.26**	0.16**	−0.01	−0.12*	--								
6	Entrepreneurship (1 = yes)	0.18	--	−0.36**	0.16**	0.03	−0.06	0.69**	--							
7	Nursing (1 = yes)	0.36	--	0.26**	−0.45**	−0.10*	−0.12**	−0.26**	−0.36**	--						
8	Veterinary Science (1 = yes)	0.46	--	0.03	0.31**	0.11**	0.16**	−0.29**	−0.44**	−0.69**	--					
9	ESE-pre-venture# (1–7)	4.08	1.13	−0.29**	0.22**	0.10*	0.13**	0.26**	0.35**	−0.29**	0.00	--				
10	ESE-people# (1–7)	4.54	1.20	−0.01	0.14**	0.06	0.11*	0.07	0.15**	−0.12**	0.00	0.66**	--			
11	ESE-finance# (1–7)	3.85	1.55	−0.23**	0.11*	0.10*	0.15**	0.21**	0.30**	−0.14**	−0.09 *	0.71**	0.63**	--		
12	OE-start-business-rc# (1–7)	4.65	1.77	−0.28**	0.28**	0.16**	0.17**	0.14**	0.24**	−0.39**	0.19**	0.51**	0.33**	0.40**	--	
13	Corporate Work Intent-rc (1–7)	3.91	1.73	−0.10*	−0.03	−0.05	0.02	0.30**	0.34**	0.04	−0.30**	0.18**	0.14**	0.21**	0.03	--
14	Entrepreneurial Intent-rc (1–7)	3.35	2.01	−0.31**	0.32**	0.17**	0.20**	0.25**	0.31**	−0.48**	0.22**	0.53**	0.29**	0.40**	0.70**	0.11**

SD standard deviation; # composite score formed by averages of contributing items; (-) minimum and maximum; n.a. not applicable as dichotomous variables; ESE entrepreneurial self-efficacy; OE outcome expectations; ** correlation is significant at the 0.01 level (2-tailed); * Correlation is significant at the 0.05 level (2-tailed); rc items recalibrated from 1–10 scale to 1–7 scale.

**Table 4:** Means and standard deviations of entrepreneurial and corporate work intention by field-of-study and gender (n = 515).

Field-of-Study	EI (s.d.)	EI (s.d.) Men	EI (s.d.) Women	CWI (s.d.)	CWI (s.d.) Men	CWI (s.d.) Women
Entrepreneurship	4.61 (1.60) ^#^	4.77	4.40	5.13 (1.49) ^#^	4.99	5.32
Nursing	2.11 (1.61) ^#^	2.37	2.08	3.99 (1.82) ^#^	4.00	3.99
Veterinary	3.81 (1.93)	4.75 *	3.52	3.35 (1.46)	3.56	3.29
All	3.35 (2.01)	3.90 *	2.95	3.91 (1.73)	4.23 *	3.80

EI entrepreneurial intention; CWI corporate work intention; * significant at the 0.001 level; ^#^ significant difference of veterinary respondents to other disciplines using Dunnett 2-sided post hoc test (*p* < 0.001).

**Table 5:** Means and standard deviations of entrepreneurial self-efficacies and outcome expectations of business start-up or ownership by field-of-study and gender (n = 515).

Field-of-Study	ESE-Pre- Venture (s.d.)	ESE-Pre- Venture (s.d.) Men	ESE-Pre- Venture (s.d.) Women	ESE-People (s.d.)	ESE-People (s.d.) Men	ESE-People (s.d.) Women	ESE-Finances (s.d.)	ESE-Finances (s.d.) Men	ESE-Finances (s.d.) Women	OE-Start Business (s.d.)	OE-Start Business (s.d.) Men	OE-Start Business (s.d.) Women
Entrepreneurship	4.85 (0.73)^##^	4.94	4.71	4.93 (0.89)	4.99	4.84	4.78 (1.14)^##^	4.81	4.75	5.50 (1.47)	5.77 *	5.12 *
Nursing	3.58 (1.29)^#^	4.35 **	3.50 **	4.25 (1.48)^#^	4.14	4.26	3.53 (1.75)	4.11	3.47	3.72 (1.72)^##^	4.26	3.66
Veterinary	4.15 (0.92)	4.46 **	4.05 **	4.60 (1.00)	4.71	4.57	3.73 (1.39)	4.26 ***	3.56 ***	5.03 (1.60)	5.59 **	4.86 **
All	4.09 (1.11)	4.51 (0.97) ***	3.88 (1.11) ***	4.57 (1.18)	4.79 (1.08) ***	4.47 (1.21) ***	3.96 (1.54)	4.55 (1.37) ***	3.67 (1.54) ***	4.62 (1.74)	5.22 (1.53) ***	4.33 (1.76)***

ESE entrepreneurial self-efficacy; OE outcome expectations; *** significant between genders at the 0.001 level; ^##, ###^ significant difference of respondents to veterinary respondents (^#^ *p* < 0.01, ^##^ *p* < 0.001).

**Table 6:** Curricula content relevant to understanding of the business of veterinary practice for veterinary respondents of this study per veterinary program (university) per year level.

Program (Length)	Early Program	Mid-Program		Late Program
University 1 * (6 years)	II. To demonstrate basic business enterprise skills applicable to the veterinarian (farm-related financial records and partial budgeting)	III. Apply business enterprise skills to scenarios (practice cash flow budget)	IV. Demonstrate an elementary knowledge of key issues in veterinary business and enterprises (e.g., veterinary business models, pricing of veterinary services, estimating, quoting)	
University 2 * (5.5 years)				V. Be able to demonstrate developing knowledge of basic practice financial management
University 3 * (5 years)	II. Understand basic concepts in veterinary economics			

II–V indicate year level in the veterinary program where II = year one and V = year five; * Royal College of Veterinary Surgeons (RCVS) accredited.

**Table 7:** Level of support for hypotheses.

No.	Hypotheses	Supported/Not
1	Veterinary science final-year students will be less interested in entrepreneurship than entrepreneurship final-year students.	Supported
2	Veterinary science final-year students will be more interested in entrepreneurship than nursing final-year students.	Supported
3	Distinct interpretable profiles of differing levels of entrepreneurial intention, entrepreneurial self-efficacy, and corporate work intention will emerge for our study population, such that potential entrepreneurial and intrapreneurial groupings of respondents will be identifiable.	Supported
4	The proportional representation of identified cluster-based profiles for entrepreneurial intention and self-efficacies will differ for veterinary respondents to entrepreneurship or nursing respondents.	Supported

## Data Availability

Data supporting reported results can be provided by contacting the primary author until all publications arising from the data set are published, after which, the data will likely be stored in a repository.
